# Supplementary material for: Molecular and Biological Investigation of Isolated Marine Fungal Metabolites as Anticancer Agents: A Multi-Target Approach
Source: Metabolites. 2023 Jan 21;13(2):162. doi: 10.3390/metabo13020162 (PMC9964656; doi:10.3390/metabo13020162)
Supplement: Supplementary file 1 [file metabolites-13-00162-s001.zip › metabolites-2122385-supplementary.pdf]

Molecular and Biological Investigation of Isolated Marine Fungal Metabolites as Anticancer Agents: A Multi-Target Approach

Hanin A. Bogari [1,†], Sameh S. Elhady [2,*,†], Khaled M. Darwish [3,*], Mohamed S. Refaey [4], Radi A. Mohamed [5], Reda F. A. Abdelhameed [6,7], Ahmad J. Almalki [8], Mohammed M. Aldurdunji [9], Manar O. Lashkar [1], Samah O. Alshehri [1], Rania T. Malatani [1], Koji Yamada [10] and Amgad I. M. Khedr [11]

[1] Department of Pharmacy Practice, Faculty of Pharmacy, King Abdulaziz University, Jeddah 21589, Saudi Arabia
[2] Department of Natural Products, Faculty of Pharmacy, King Abdulaziz University; Jeddah 21589, Saudi Arabia
[3] Department of Medicinal Chemistry, Faculty of Pharmacy, Suez Canal University, Ismailia 41522, Egypt
[4] Department of Pharmacognosy, Faculty of Pharmacy, University of Sadat City, Sadat City 32897, Egypt
[5] Department of Aquaculture, Faculty of Aquatic and Fisheries Sciences, Kafrelsheikh University, Kafrelsheikh, Egypt
[6] Department of Pharmacognosy, Faculty of Pharmacy, Galala University, New Galala 43713, Egypt
[7] Department of Pharmacognosy, Faculty of Pharmacy, Suez Canal University, Ismailia 41522, Egypt
[8] Department of Pharmaceutical Chemistry, Faculty of Pharmacy, King Abdulaziz University, Jeddah 21589, Saudi Arabia
[9] Department of Clinical Pharmacy, College of Pharmacy, Umm Al-Qura University, P.O. Box 13578 Makkah 21955, Saudi Arabia
[10] Garden for Medicinal Plants, Graduate School of Biomedical Sciences, Nagasaki University; Bunkyo-machi 1-14, Nagasaki 852-8521, Japan
[11] Department of Pharmacognosy, Faculty of Pharmacy, Port Said University, Port Said 42526, Egypt

Detailed Experimental Procedure

*Fermentation, Extraction, Isolation, and Purification of Compounds **1**–**3***

Fungus *P. chrysogenum* strain S003 was cultured through incubation on a static condition at 25°C for 30 days in 3000 mL 20 conical flasks containing the liquid medium (750 mL/flask) composed of Czapek yeast media ($NaNO_3$ 3.0 g, K_2HPO_4 1.0 g, $MgSO_4{\cdot}7H_2O$ 0.5 g, KCl 0.5 g, $FeSO_4$ 0.01 g, yeast extract 5.0 g, sucrose 30.0 g and NaCl 20.0 g, dissolved in distilled water 1,000 mL). The fermented whole broth (15 L) was filtered after addition of acetone through cheesecloth to separate into filtrate and mycelia. The filtrate was concentrated in vacuo to about 2 L and then extracted three times with EtOAc which was further evaporated in vacuo to afford an EtOAc extract, while the mycelia were extracted three times with methanol. The methanol extract was combined with the EtOAc extract based on TLC monitoring followed by evaporation till dryness. The residue was dissolved in methanol followed by partitioning with n-hexane and evaporation till dryness to give methanol extract (C-E = 4.2 g) and n-hexane extract (C-H = 5 g).

The crude extract (C-E) was purified through chromatography over Sephadex LH-20 column and eluted with $CHCl_3$: MeOH (1:1) to yield 10 sub-fractions (C-E-1 ~ C-E-10) based on TLC analysis. Fraction C-E-4 (0.5 g) was subjected to SiO_2 column elution with a gradient of increasing EtOAc in *n*-Hexane to afford 6 fractions (C-E-4-1 ~ C-E-4-6). Fraction C-E-5 (165 mg) was subjected to fractionation over Sephadex LH-20 column and eluted with $CHCl_3$: MeOH (1:1) to yield 4 sub-fractions (C-E-5-1 ~ C-E-5-4). Fraction

C-E-5-2 (127 mg) was finally purified over silica gel column using *n*-Hexane: EtOAc (9:1 ~ 1:9) gradient elution to give meleagrin (MEL) (58 mg). Fraction C-E-6 (390 mg) was chromatographed over silica gel column using *n*-Hexane: EtOAc (2:8 ~ 0:10) gradient elution to yield 7 sub-fractions (C-E-6-1 ~ C-E-6-7). Fraction C-E-6-5 (27 mg) was chromatographed over Sephadex LH-20 column and eluted with MeOH isocratic elution to afford 5 sub-fractions (C-E-6-5-1 ~ C-E-6-5-5). Fraction C-E-6-5-3 (20 mg) was chromatographed over Octadecylsilane (ODS; Nacalai, Inc., Kyoto, Japan, (0.5 x 10 cm, id)) silica gel column using MeOH: H_2O (6.5:3.5) isocratic elution followed by silica gel column using $CHCl_3$: MeOH (97:3) to give roquefortine C (ROC), (5 mg) and isoroquefortine C (ISO) (9 mg).

Instrumentation

NMR spectra were recorded using Varian Unity-Plus 400 spectrometer (Palo Alto, CA, USA) operating at 400 MHz for 1H and 100 MHz for ^{13}C and Bruker Avance DRX-500 spectrometer at 500 MHz ^{1}H-NMR and 125 MHz ^{13}C-NMR, Germany. The FAB-MS spectra were recorded on a JMS 700N spectrometer (JEOL Ltd., Tokyo, Japan) in positive ion mode.

№.	Contents	Page
**Figure S1.**	^{1}H-NMR spectrum of compound **1**	3
**Figure S2.**	^{13}C-NMR spectrum of compound **1**	4
**Figure S3.**	NOESY spectrum of compound **1**	4
**Figure S4.**	^{1}H-NMR spectrum of compound **2**	5
**Figure S5.**	^{13}C-NMR spectrum of compound **2**	5
**Figure S6.**	NOESY spectrum of compound **2**	6
**Figure S7.**	^{1}H-NMR spectrum of compound **3**	6
**Figure S8.**	^{13}C-NMR spectrum of compound **3**	7

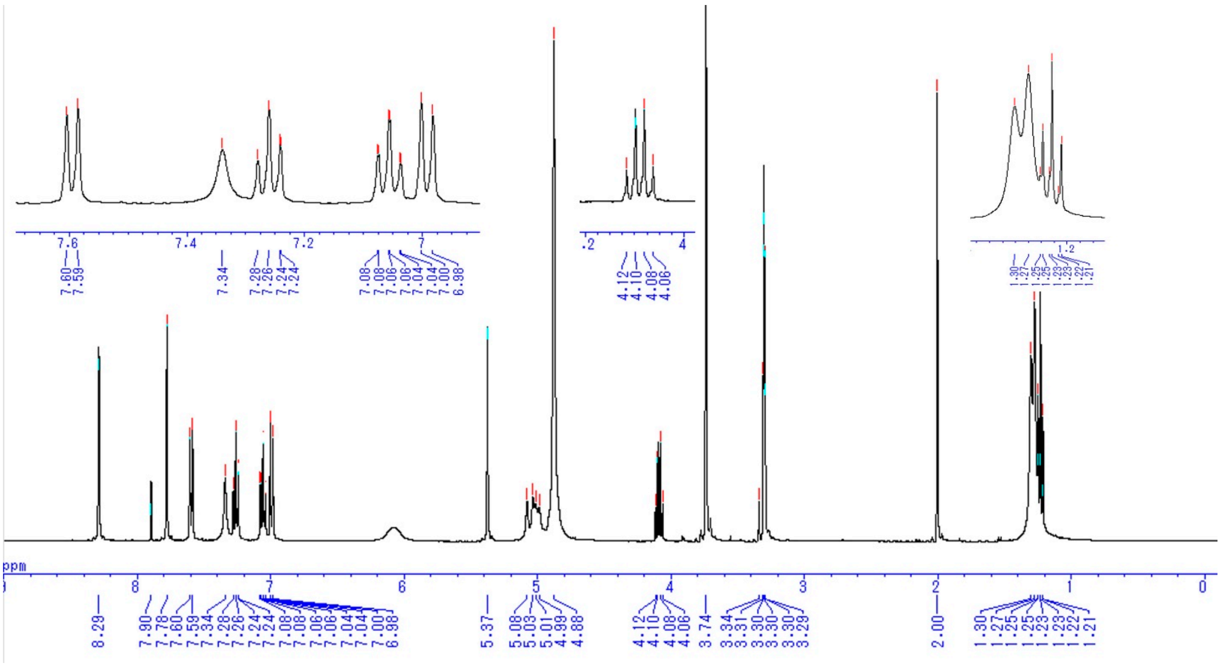

Figure S1. ^{1}H-NMR spectrum of compound **1** ($CDCl_3$, 400 MHz)

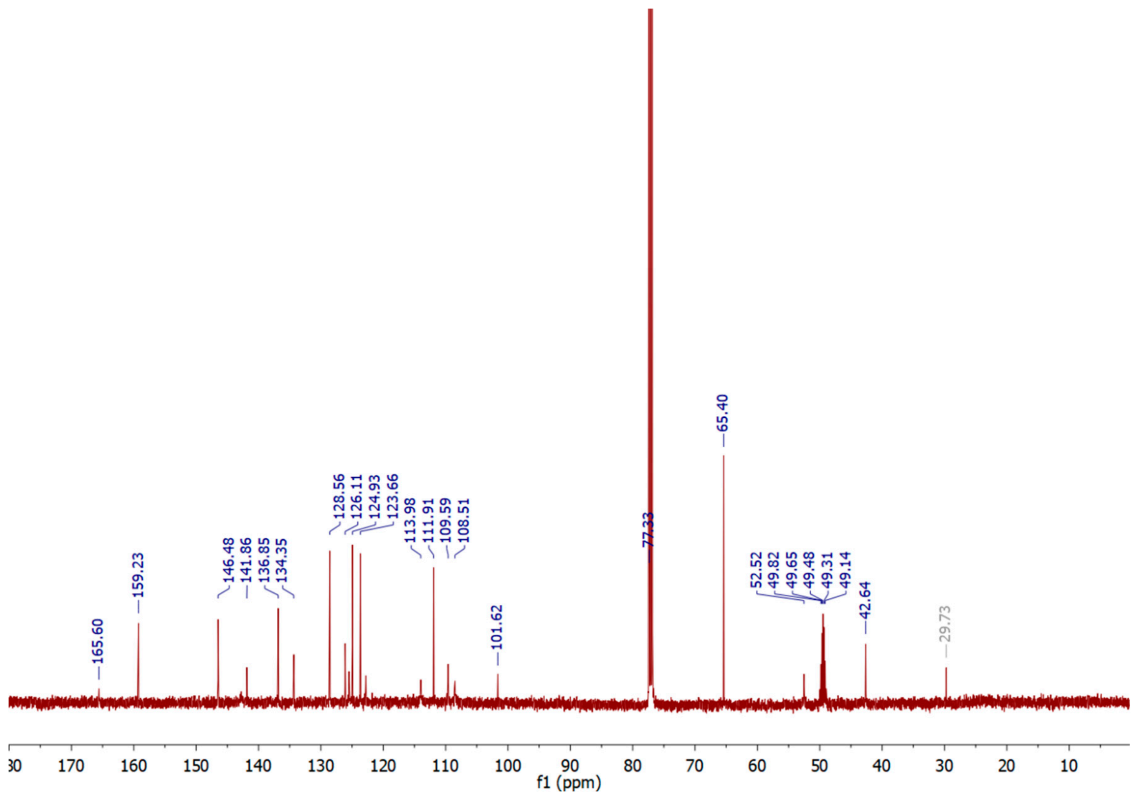

Figure S2. ^{13}C-NMR spectrum of compound **1** (CD_3Cl: CD_3OD (1:1), 125 MHz)

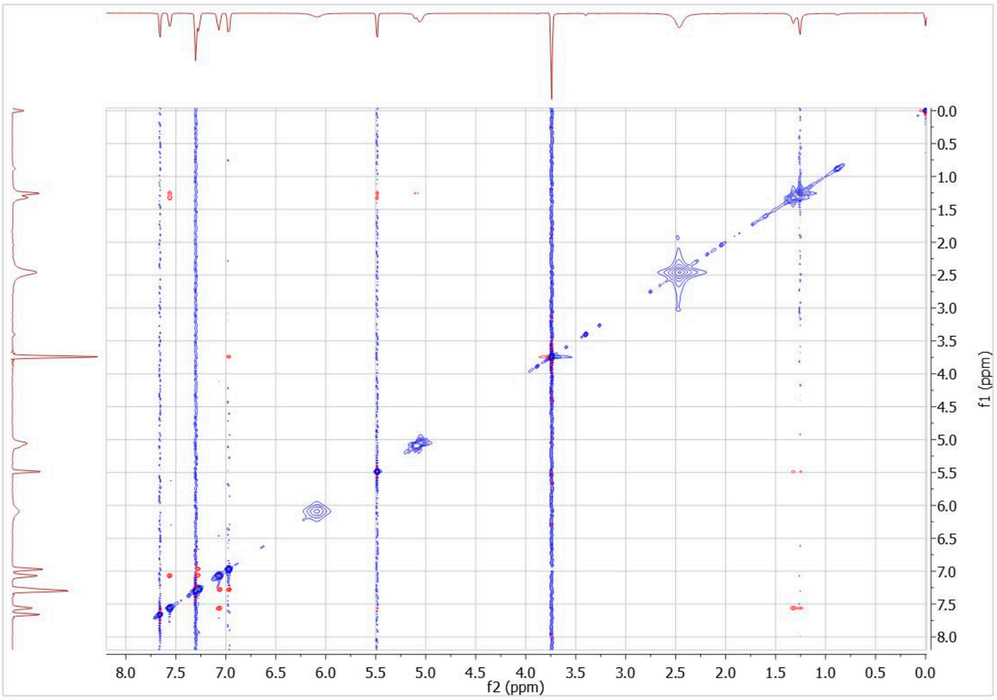

Figure S3. NOESY spectrum of compound **1**

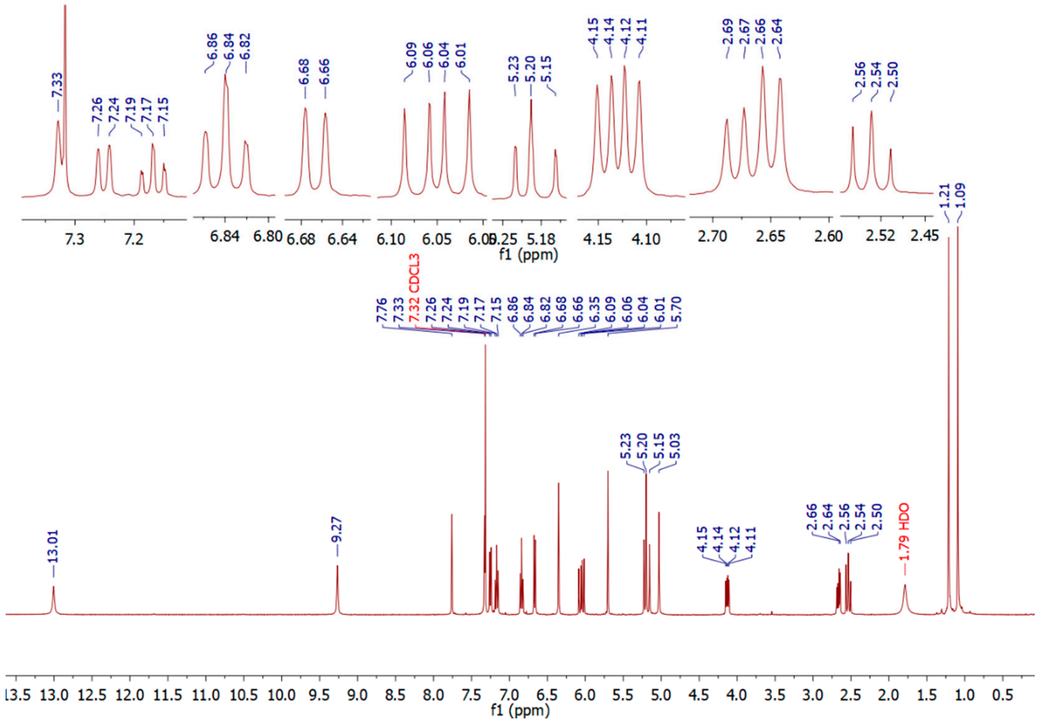

Figure S4. ^{1}H-NMR spectrum of compound **2** ($CDCl_3$, 400 MHz)

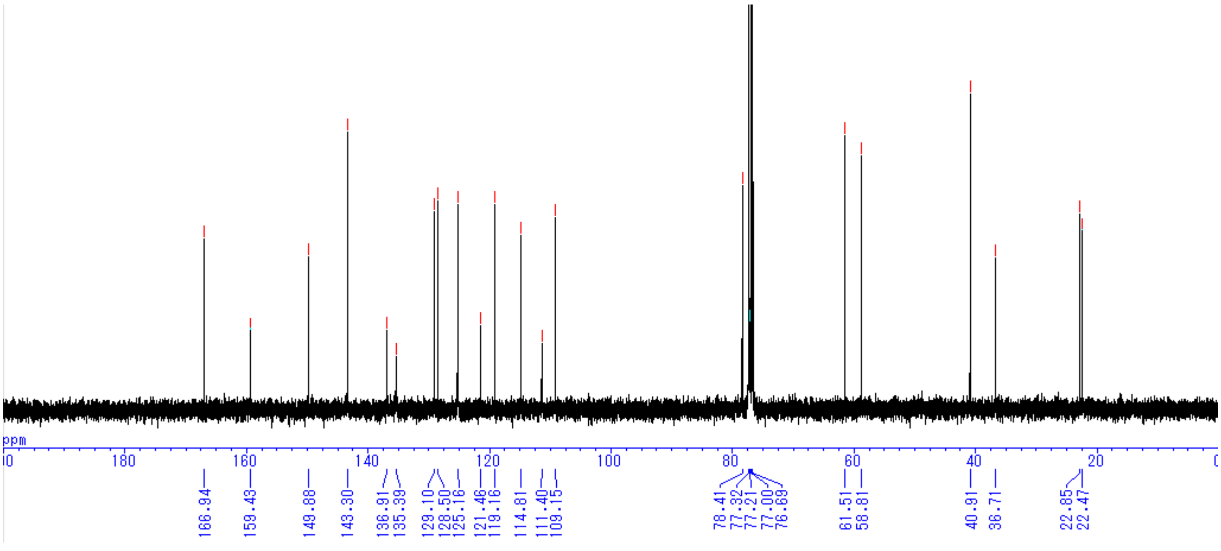

Figure S5. ^{13}C-NMR spectrum of compound **2** ($CDCl_3$, 100 MHz)

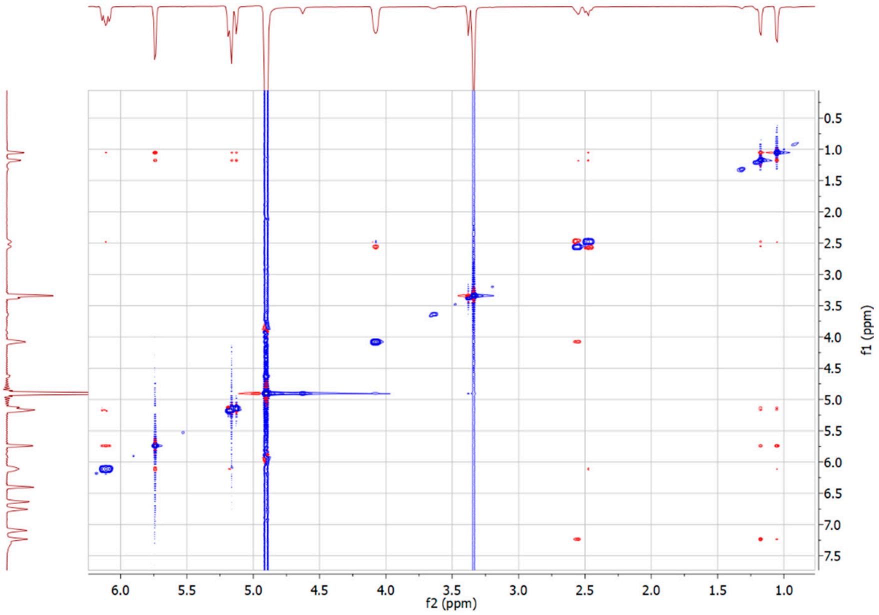

Figure S6. NOESY spectrum of compound **2**

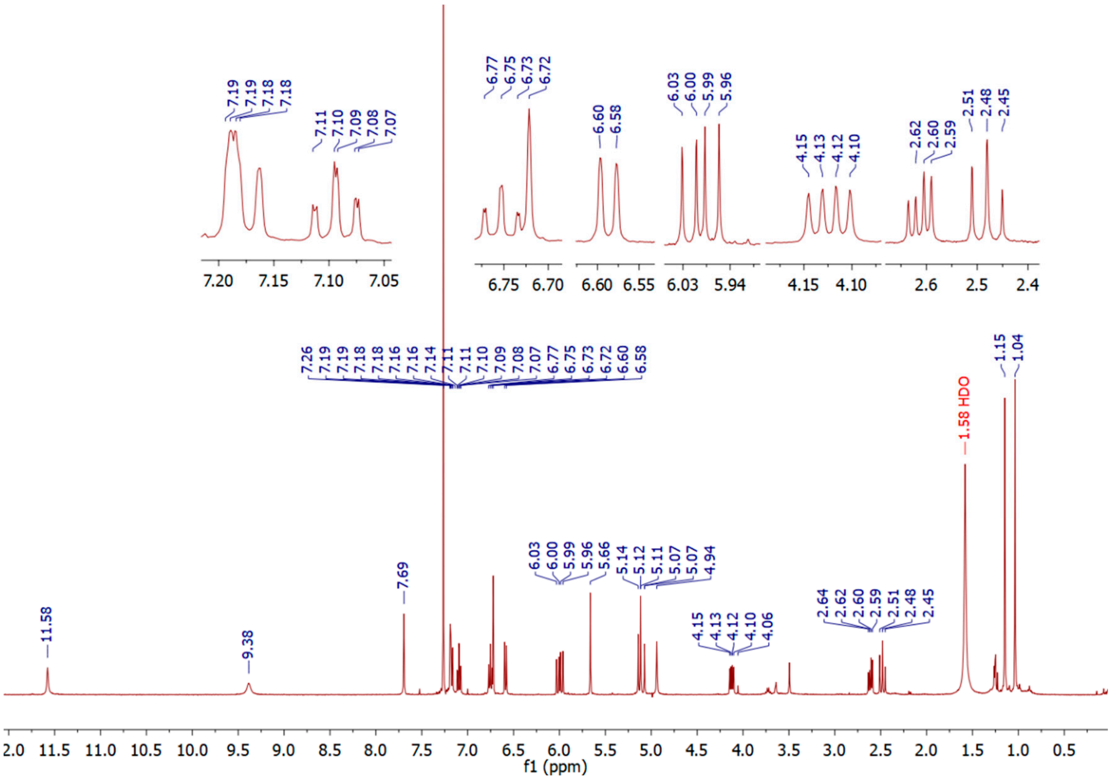

Figure S7. ^{1}H-NMR spectrum of compound **3** ($CDCl_3$, 400 MHz)

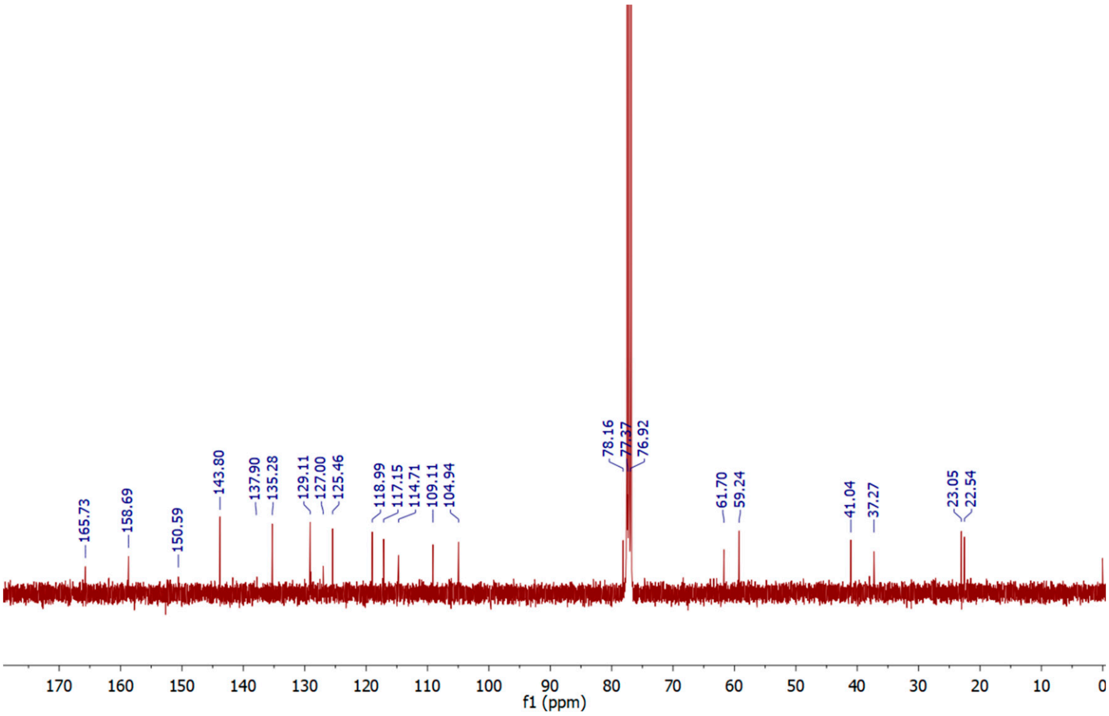

Figure S8. ^{13}C-NMR spectrum of compound **3** ($CDCl_3$, 100 MHz)
